# Network analysis reveals why *Xylella fastidiosa* will persist in Europe

**DOI:** 10.1038/s41598-017-00077-z

**Published:** 2017-03-06

**Authors:** Giovanni Strona, Corrie Jacobien Carstens, Pieter S. A. Beck

**Affiliations:** 1 European Commission, Joint Research Centre, Directorate D - Sustainable Resources, Bio-Economy Unit, Via Enrico Fermi 2749, 21027 Ispra, Italy; 2 School of Mathematical and Geospatial Sciences, RMIT University, Melbourne, Victoria 3000 Australia

## Abstract

The insect vector borne bacterium *Xylella fastidiosa* was first detected in olive trees in Southern Italy in 2013, and identified as the main culprit behind the ‘olive quick decline syndrome’. Since then, the disease has spread rapidly through Italy’s main olive oil producing region. The epidemiology of the outbreak is largely unstudied, with the list of *X. fastidiosa* hosts and vectors in Europe likely incomplete, and the role humans play in dispersal unknown. These knowledge gaps have led to management strategies based on general assumptions that require, among others, local vector control and, in certain areas, the destruction of infected plants and healthy ones around them in an attempt to eradicate or halt the spreading pest. Here we show that, regardless of epidemiological uncertainties, the mere distribution of olive orchards in Southern Italy makes the chances of eradicating *X. fastidiosa* from the region extremely slim. Our results imply that Southern Italy is becoming a reservoir for *X. fastidiosa*. As a consequence, management strategies should keep the prevalence of *X. fastidiosa* in the region as low as possible, primarily through vector control, lest the pathogen, that has also been detected in southern France and the island of Mallorca (Spain), continues spreading through Italy and Europe.

## Introduction

Olive farming in Southern Italy’s Puglia region dates back to pre-Roman times^1^ and currently accounts for nearly 40% of Italy’s olive oil production (434,000 tons/yr between 2011 and 2014)^2^. Since 2013, this millenary tradition is threatened by Europe’s first major outbreak of *X. fastidiosa* ^3^, a pathogen that has a long phytosanitary history in the Americas as a pest of grapevine and citrus trees^4^. During the outbreak, the bacterium, which was most likely introduced from Central America^3^, encountered in Puglia a large percentage of its many already known host plants^5^ and, in olive trees, a new suitable host^6^. Here, we investigate how much the landscape layout in Puglia contributed to the spread of *Xylella fastidiosa*, and what clues it holds on the options for eradicating or containing the epidemic.

The list of insects that can theoretically serve as vectors for *X. fastidiosa* in Europe, and transmit it to plants during xylem-fluid feeding, includes dozens of species^7^. The apparent vector in the ongoing *X. fastidiosa* outbreak in the olive orchards of Southern Italy is the meadow splittlebug, *Philaenus spumarius* (Linnaeus, 1758), which is also the most abundant xylem-fluid feeding hemipteran in the area^6, 8^. Despite moving preferentially by jumping, adult splittlebugs can fly. While there is little specific information available on *Philaenus spumarius*, other spittlebug species can fly more than 900 meters, with their flight duration and speed indicating a maximum single-flight distance of up to 3 km^9^. Field measurements for *P. spumarius* itself, have shown shorter dispersal distances, with specimens moving for about 100 meters per day^10^. Since *P. spumarius* adults can survive up to 4–6 months (from late spring to late fall^10^), this daily movement distance suggests, very conservatively, that an individual may travel up to hundreds or thousands of meters, particularly if aided by wind^10^. Even if the probability of an individual spittlebug traveling this distance is very low, the high local abundance of *P. spumarius* makes such ‘long-distance’ dispersal likely.

On the basis of these considerations, we assumed that *X. fastidiosa* can be transported by a vector between host plants that are up to 1 km apart. This cautious assumption does not consider the possibility of there being better fliers among the other potential *X. fastidiosa* vectors, i.e. all xylem-feeding insects in the region. Nonetheless, we also considered even more conservative scenarios that reduced the literature-based travel distance estimate for *P. spumarius* by half, to 500 m.

**Figure 1:**
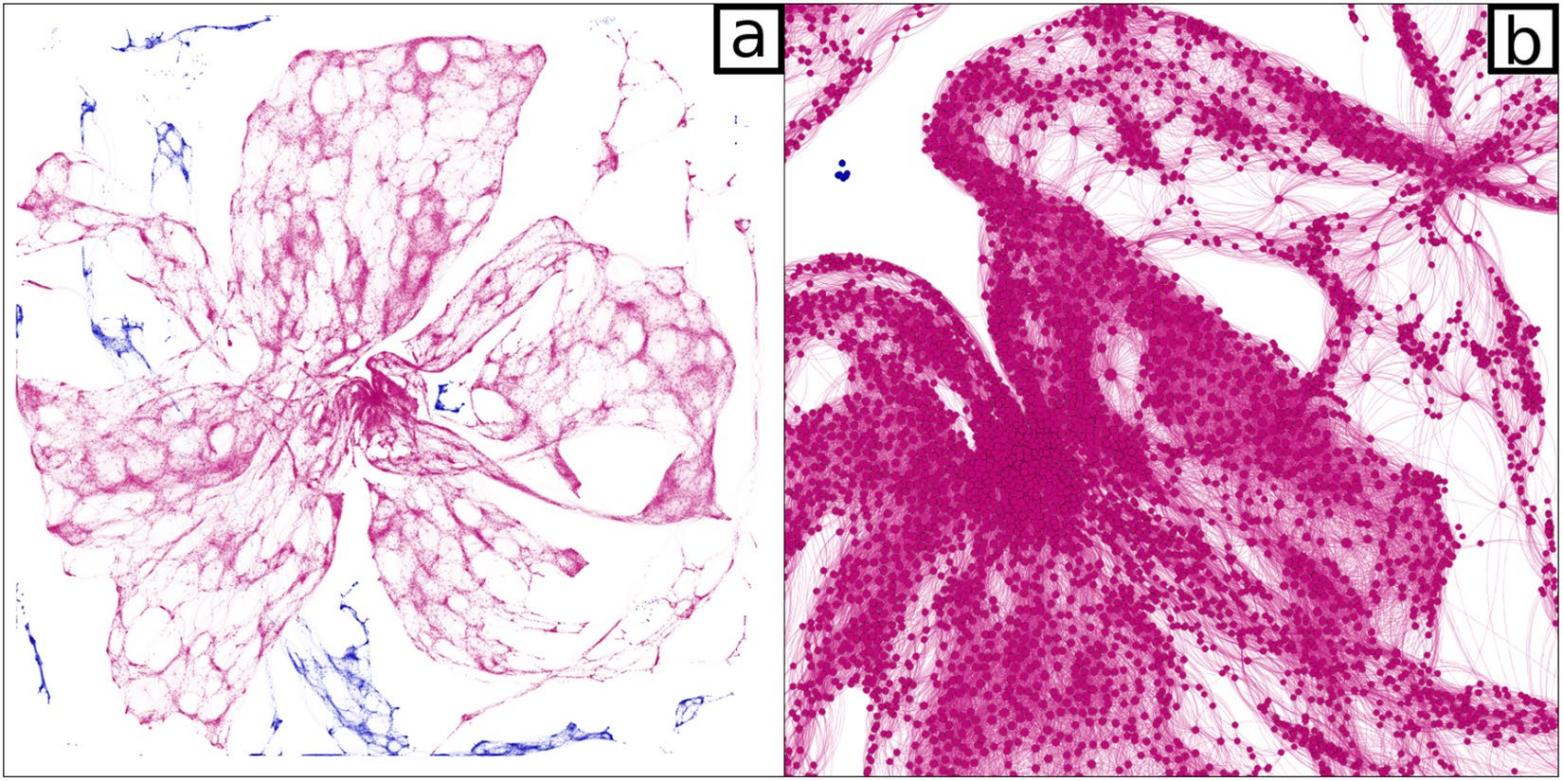
The network of olive orchard in Puglia. (**a**) Full view. (**b**) 10X zoom of the central region. Dots represent olive orchards, while links join all pairs of orchards at a distance of less than 1 km, depicting possible pathways of spread for Xylella fastidiosa. Edges and nodes belonging to the largest connected component (LCC) of the network are colored in magenta, while all other nodes and edges are colored in blue.

Using a high resolution map of olive orchards, we generated a network of potential *X. fastidiosa* infection pathways in Puglia by connecting all pairs of olive orchards (i.e. nodes in the network) that are within the vector’s reach, i.e. less than 1 km (μ) apart (with the distance measured between the two closest sides of the two orchards). This network, which consists of 60,886 nodes (i.e. 99.8% of mapped orchards) and 869,781 edges (Fig. 1), deliberately ignores the real possibilities of human aided long distance dispersal, of multiple, and potentially better-flying, vectors, and of other plants capable of hosting *X. fastidiosa* growing between olive orchards. Thus, it approaches a best-case scenario for the *X. fastidiosa* epidemic, since taking into account any of these possibilities would dramatically increase the number of possible infection pathways (i.e. network edges).

**Figure 2:**
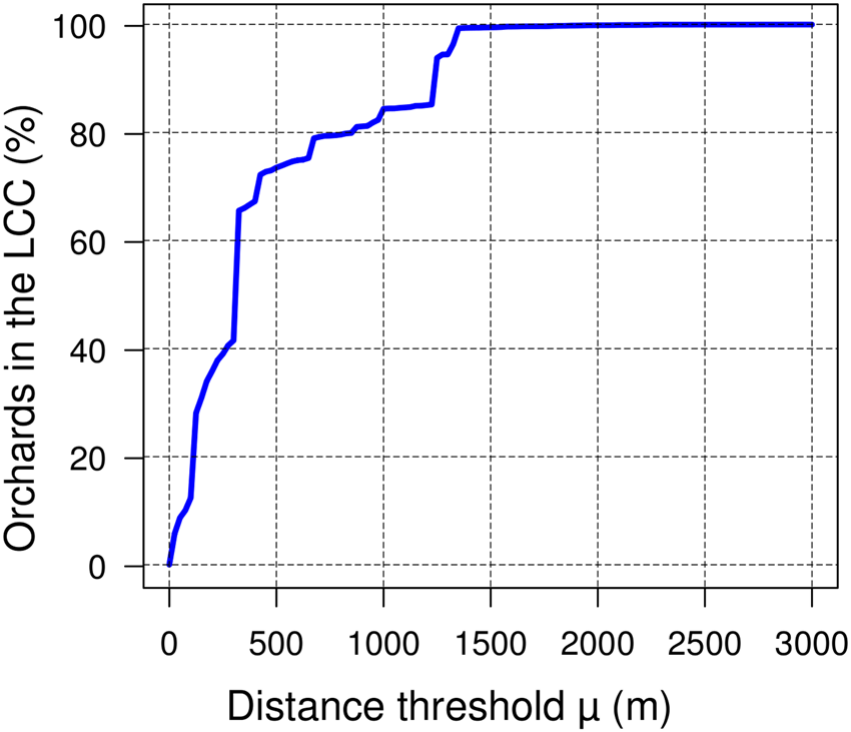
Relationship between the distance threshold μ and the percentage of orchards in the largest connected component (LCC) of the corresponding network. When μ exceeds 1,500 m more than 99.9% of the orchards belong to the LCC and the entire network is almost connected.

We investigated the network’s structure to provide a quantitative perspective, with minimal reliance on biological assumptions and independent from control measures^11^, on the effort required to manage the *X. fastidiosa* outbreak in Southern Italy. First, we evaluated how many orchards can be potentially reached by the infection providing the bacterium can only move between connected orchards (being those close enough to permit an infected *P. spumarius* individual to move from one to another). For this, we identified the largest connected component (LCC) of the olive orchard network, which is the largest set of nodes for which at least one path to any other node in the set exists. In the network obtained using the 1 km threshold, the LCC includes 85% (51,499) of all nodes (Figs 1 and 2). In the much more conservative scenario, where the threshold for two orchards to be connected is halved (μ = 500 m), the LCC still reaches a comparable size (73%). Increasing the threshold by 35% (μ = 1,350 m) is sufficient to create a network where >99% of nodes are connected (Fig. 2). In all cases, most of the olive orchards in the currently infected zone^11^ belong to the LCC (99.99% in the case of the network based on μ = 1 km, Supplementary Fig. S1; see http://webapps.sit.puglia.it/freewebapps/MonitoraggioXFSintesi/ for an updated map of confirmed cases of *X. fastidiosa* infection in Puglia). This means that, even under the highly conservative assumption that orchards that are more than 500 m from an infected one are not at risk of infection, less than 30% of orchards in the region can be considered ‘safe’ (i.e. not reachable by the disease because isolated from the currently infected portion of the network). The percentage of ‘safe’ nodes rapidly approaches 0 when less conservative scenarios are considered.

The fact that the LCC extends to almost the entire network, even when μ is set very conservatively, is the result of the high density and close proximity of the olive orchards in Puglia, with the distance between an orchard and its nearest neighbor (measured between the two closest points on the orchards’ perimeters) being extremely small, averaging 57 m ± 124.8 m (s.d.) and no more than 4,061 m (Fig. S2). This reveals how the topology of the original olive orchard network gives *X. fastidiosa* the potential to eventually reach most parts of Puglia, and establish itself in Southern Italy, as *X. fastidiosa* subsp. *fastidiosa* has done in the USA^12^. Given this premise, we used further network analysis to shed light on the level of intervention required to prevent this from happening.

We assessed the effort needed to ‘immunize’ the olive orchard network against a poorly understood epidemic such as that of *X. fastidiosa*. Note that, here, we use the term ‘immunization’ in the sense of network theory rather than plant pathology, referring to strategies aimed at identifying and removing specific nodes from a network in order to break it apart into relatively small, isolated fragments that prevent a local infection from becoming a pandemic^13, 14^. Thus, in this context, the ‘removal’ of a node should not necessarily be interpreted as the full clearance of an orchard, but as any measure that keeps it uninfected.

Identifying an efficient strategy to immunize a network is a problem that has received much attention, leading to very efficient, but often counterintuitive, criteria of node removal^14^. We applied such criteria to the olive orchard network, and recorded the fraction of nodes remaining in the LCC after the sequential removal of nodes. The amount of nodes one needs to remove in order to reduce the size of the LCC provides a robust indicator of the potential efficacy of network immunization.

**Figure 3:**
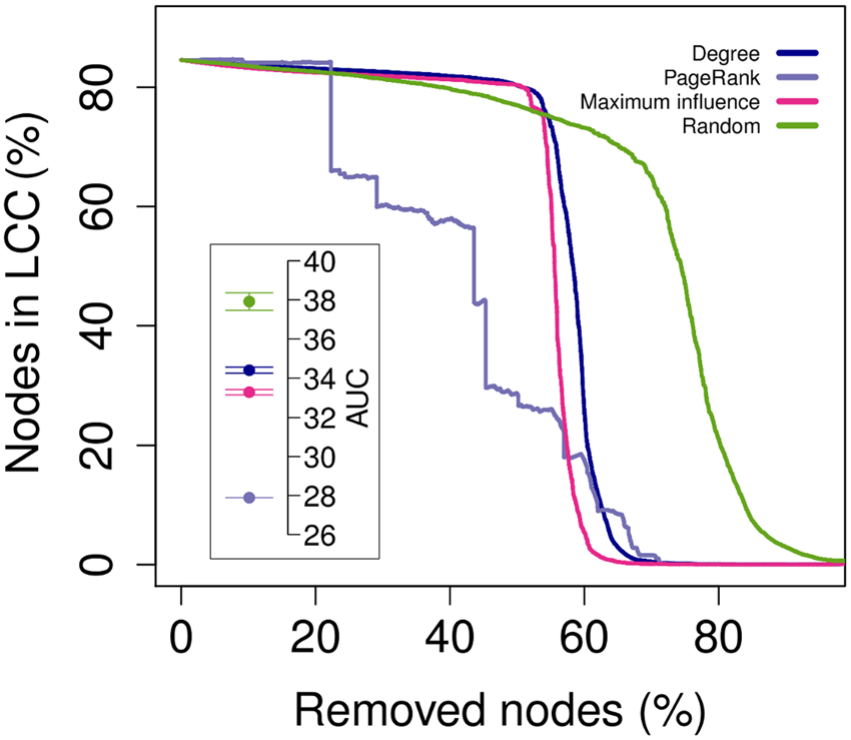
Effort needed to immunize the olive orchard network. Lines show the fraction of nodes remaining in the largest connected component (LCC) of the olive orchard network after progressive node removal according to different criteria (see Methods). The solid lines interpolate the average values of 100 replicates. The inner box reports the average areas under the curve (over 100 replicates) for each removal criterion, rescaled between 0 and 100, with error bars indicating standard deviations.

Removing nodes at random had a poor immunization effect on the olive orchard network, with a very slow reduction of the fraction of nodes in the LCC, and the network only breaking apart after 80% of nodes had been removed (Fig. 3). By comparison, the most efficient criterion (PageRank^15^) reduced the effort needed for an effective immunization considerably. Yet, even in this case, reducing the LCC by half required the removal of more than 40% of the nodes. In an experiment on a less connected network (μ = 500 m), at least 15% of nodes (equivalent to nearly 9,000 orchards) had to be removed to obtain the same effect (Supplementary Fig. S3). The resistance of the network to immunization, and the fact that most olive orchards in Puglia are hundreds of years old, indicate that preventing the current epidemic would have required a profound reshaping of both Puglia’s age-old cultural landscapes^1^, and of the public perception of their vulnerability.

**Figure 4:**
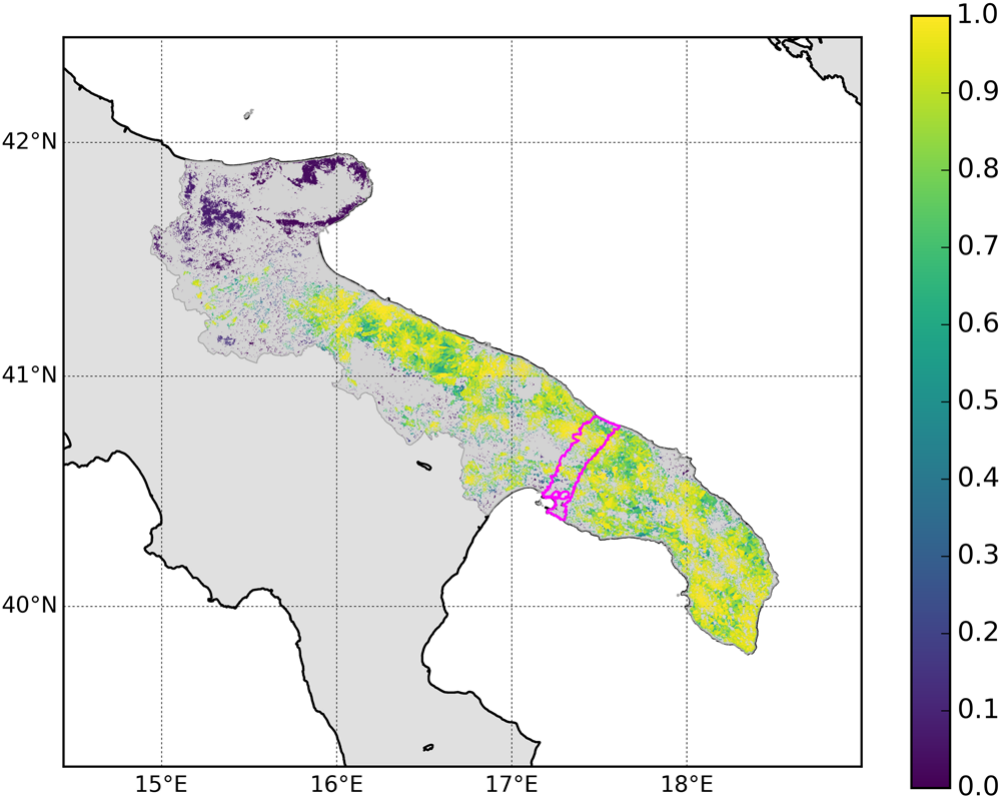
Relative importance of olive orchards as spreaders of *Xylella fastidiosa* in Puglia. Colors show the probability (averaged over 1000 simulations and rescaled between 0 and 1) of an orchard becoming infected with *X. fastidiosa* in simulations of the bacterium becoming endemic in the Puglia region. Dark grey areas are other land cover types in Puglia, while light grey areas were not analyzed. The magenta line delineates the current buffer zone that is intensely monitored and aims to prevent *X. fastidiosa* from spreading north. The map was generated using the Python Basemap Matplotlib Toolkit (http://matplotlib.org/basemap/).

Our findings highlight the need for a management strategy that keeps the prevalence of *X. fastidiosa* low in order to save at least part of the local olive oil production, and reduce the role of the region as an Italian and European reservoir for the pathogen. The management procedures currently adopted in Puglia call, among others, for intensified monitoring and the removal of infected plants in a 20 km wide zone at the edge of the infected area, as well as the removal of host plants surrounding infections in a 10 km wide buffer zone beyond it^11^ (Fig. 4). This buffer aims to prevent *X. fastidiosa* from spreading north and reaching Southeast Italy, another major (>40%) contributor to Italy’s olive oil production^2^. Such a containment barrier is efficient for disease control provided it can be maintained impenetrable to the pathogen, but, conversely, a single jump of the pathogen across the barrier can compromise it^16^. This raises the question whether specific and robust priority targets for management, other than all susceptible plant species in the buffer zone, can be identified. Our analysis investigated whether a strategy that considers the topological properties of the olive orchard network could strengthen and focus disease control efforts.

We found that the topology of the olive orchard network in Puglia favors persistence of the *X. fastidiosa* epidemic, and complicates management prioritization. Ranking the orchards based on their contribution to the *X. fastidiosa* spread once it becomes, as is expected, endemic^17^, showed that most olive groves in Puglia can act as influential spreaders of the pathogen and should, in theory, be kept under strict control (Fig. 4). Nodes with limited spreading influence are predominantly in the northeast of Puglia, where orchards are sparser and less connected to the rest of the network. Coincidentally, climatic conditions in this area might also be less favourable for the disease^18–20^.

The phytosanitary buffer zone currently stretches across the northern end of Puglia’s main peninsula^21^. This positioning limits the size of the area subject to most intense surveillance and management, and aims to prevent the spread of *X. fastidiosa* beyond the peninsula. Our findings, however, show that the layout of Puglia’s agricultural landscape does not facilitate the eradication measures in this particular zone, as it is located in an area where nodes have high spreading influence (Fig. 4).

The difficulties we document in either immunizing the olive orchard network or identifying priority targets to manage the epidemic spread of *X. fastidiosa* in Puglia are in part an obvious consequence of the spatial nature of the olive orchards’ network^22^. Since the existence of a link between two nodes in the network reflects their respective location in the real world, the number of links per node is constrained by the spatial density of orchards, and by the chosen threshold distance (μ). At the same time, however, our results are driven by a combination of peculiarities of the Puglia olive orchard network that are not necessarily shared by all geometric networks^22^. Real-world geometric networks often follow a power law degree distribution, where many nodes have few connections, while a few nodes have a disproportionately high number of connections and act as hubs of primary importance for the diffusion of disease or information^23^. Here, however, the elongated geometry of the Puglia region, together with the very high and relatively uniform density of olive orchards, leads to a network with very high redundancy, in which most nodes have more than 35 connections and obvious targets for disease spread control do not emerge (see Supplementary Fig. S5).

As we demonstrated, accounting for the structure of infection networks can orient management strategies by improving understanding of infectious disease outbreaks even when epidemiological parameters are uncertain^24^. Here, network topology revealed that most of the orchards in Puglia are within reach of the *X. fastidiosa* epidemic, and may promote the spread and persistence of the pathogen. As a consequence, eradicating the disease from the region would require an outsize effort involving most olive crops. Our analyses suggest that containing *X. fastidiosa* exclusively through host plant control would require extensive clearcutting, and imply that vector control measures are critical. The abundance of *P. spumarius* in the region^6^, the presence of several other potential vectors^7^, and the redundancy of infection pathways suggest that only a massive use of generic insecticide can address the problem rapidly. However, this does not appear a viable option: in addition to its unpredictable, and possibly dramatic, environmental consequences^25^, such large-scale insecticide use is also controversial with local farmers, who are reluctant to risk compromising the quality of olive crops^26^. Instead, milder vector control forms, such as those currently supported by local authorities^27^, including mechanical control of juveniles by removing shrubs and the use of pesticides targeting pre-infective adults of specific xylem sap-feeders, could help counteract the ongoing spread. Other longer-term management strategies could consider replacing uprooted trees with resistant varieties^26^, and research novel techniques for pest control^28^.

Despite the large knowledge gaps on the epidemiology and potential for control of *X. fastidiosa* in Europe, our analysis of the topology of the olive orchard network in Puglia and the current state of the *X. fastidiosa* outbreak there shows that Southern Italy is becoming a reservoir for the pathogen. Efforts to reduce the prevalence of *X. fastidiosa* in the region are fundamental to lower the risk that the infection spreads to olive crops in other Italian areas and Mediterranean countries. Nevertheless, since a complete eradication of *X. fastidiosa* from Puglia appears unlikely, and new introductions of infected plants from outside Europe cannot be entirely excluded, it might only be a matter of time before we will face new outbreaks. Furthermore, such new outbreaks might have an impact beyond olive farming, since several other economically valuable species in the Mediterranean are capable of hosting *X. fastidiosa* ^5, 7^. Although such scenarios might play out faster than research can provide remedies, network analysis can improve preparedness by elucidating opportunities, challenges, and priorities for management actions.

## Methods

### Building the networks

A high-resolution (1:10,000) digital map of olive orchards in the Puglia region was obtained from the regional geographic information system portal^21^. It is based on a soil usage map created from orthophotos with pixels of 50 cm and taken in 2011. The map includes 61,035 polygons, each of them corresponding to a distinct olive orchard. We identified the minimum distance between each polygon and all the neighboring polygons within a radius of 5 km. A. *csv* file including all these pairwise distances can be downloaded from the Joint Research Centre Data Catalogue (http://data.jrc.ec.europa.eu/). The distance between two polygons was measured as the linear distance between the two closest points of the polygons’ perimeters. As opposed to measuring the distance between polygon centroids, this method avoids the inflation of distances due to the area a polygon. We then used the measured distances to build several networks where we linked any two orchards within a given threshold distance (μ). In particular, we built 120 networks with μ varying between 0 and 3,000 meters (with an increment of 25 meters). Each one of these networks maps the potential infection pathways for *Xylella fastidiosa* under different hypotheses about the dispersal ability of the vector. In this scenario, each orchard in the region corresponds ideally to an ‘island’, with the selected μ indicating how far a ‘colonizer’ (i.e. an infected vector) can travel from one island to another.

As discussed in the main text, we focused our analyses on the network obtained using μ = 1 km, which is the best-estimated one emerging from our literature review. In the following, if not differently specified, we refer to this network. Isolated nodes (i.e. orchards not having neighbors within the threshold distance) were excluded from the networks. In the case of μ = 1, this led to the exclusion of only ~0.2% of the orchards reported in the high-resolution map.

For each network, we recorded various topological properties, including the number of nodes (i.e. included olive orchards) and edges (i.e. potential infection pathways), the average node degree (i.e. the average number of links per node, representing the average number of orchards within the threshold distance from a focal node), the number of connected components (i.e. all independent sets of nodes having links within but not between groups), and the size of the largest connected component.

### Network immunization

We evaluated the possibility of ‘immunizing’ the network (i.e. breaking it apart into small isolated subnetworks) by progressively removing nodes and recording, at each removal step, the fraction of nodes remaining in the largest connected component of the network. We used three different criteria to identify which node to remove at each step, namely node degree, PageRank^15^ and collective influence (*CI*)^14^. This last criterion can be extremely efficient, permitting, in several real world cases, to immunize a network removing much fewer nodes than other available methods. The *CI* of a node *i* is calculated as the sum of d(*j*)-1 for each node *j* in the *k*-neighborhood of the focal node, multiplied by d(*i*), with d(*x*) being the degree of node *x*. The *k*-neighborhood of node *i* is the set of all nodes in the network having shortest path to *i* lower or equal to *k* (i.e. the set of nodes reachable from *i* in no more than *k* steps moving from a node to another). Following the authors’ recommendation, we set *k* (the parameter ℓ in equation 5 of Morone and Makse^14^) to a conservative value of 5 (the higher *k* the better, with *k* = 3 already permitting to reach top performances).

For all the three criteria, at each step we first chose the node with the highest target value (degree, PageRank, CI), then removed it, and finally recalculated the target value for all nodes. We reiterated the procedure until no node was left in the network. To resolve ties, we replicated the entire disassembling procedure (for each criterion) 100 times, picking at random – in case of equalities - one among all nodes having the highest value. In addition to these three criteria, we disassembled the network 100 times by removing nodes in random order. Besides providing a reference to compare with targeted removal criteria (i.e. degree, PageRank and CI) with, the random removal procedure has, in the case of *Xylella* outbreak in Puglia, a more profound meaning, since it could be loosely representative of the ongoing removal procedures, which were developed based on concepts other than network properties.

### Evaluating nodes’ importance as potential epidemic spreaders

To evaluate the relative importance of nodes (i.e. olive orchards) as ‘spreaders’ we ran 1,000 Susceptible-Infected-Susceptible (*SIS*) models^13^ on the network. It is important to clarify that the application of the epidemic model was only functional to quantify node spreading importance, and was by no means intended a realistic representation of the ongoing *Xylella* spread. Thus, we are not claiming that the *SIS* is the most suitable model to represent the outbreak, nor that selected infection and recovery probabilities are accurate epidemic parameters. As a matter of fact, the many uncertainties about the epidemiological behavior of *X. fastidiosa* on olive trees make it very difficult even an attempt at model parametrization. Our goal here was to simulate “dummy” epidemics on the network in order to identify which nodes are more likely to be infected with high frequency on the solely basis of network topology, thus occupying key positions in the network to keep the disease running^17^. Thus, the concept of ‘recovering susceptibility’ that is at the core of the *SIS* model is used here without any reference to real world situations (such as, for example, replanting healthy trees).

In the application of a *SIS* model to a network, at each step of the epidemic simulation, the infection passes from infected nodes to any of their neighbors with probability *β*. With probability *λ*, any infected node turns back to the susceptible state^13, 17^. Computationally, simulation starts with a few infected nodes. At each step, the script iterates over all infected nodes at that time. Then, it sub-iterates over all the neighbors of an infected node *i*, and each neighbor of *i* gets infected with probability *β*. Once the iterations over the neighbors of node *i* is completed, node *i* recovers from infection and becomes again susceptible with probability *λ*.

In this model, the epidemic tends to reach the endemic status, that is a dynamic equilibrium where the number of infected nodes turning back to susceptibility compensates the number of new infections^17^. The nodes identified as key for the epidemic, are then those having the highest probability of being infected at a given moment (*t*) in the simulation, ρ_i_(*t* → *∞*) (17). In principle, the choice of the *β* and *λ* parameters has little importance, since it affects the average number of infected nodes at the endemic state, while it should have little effect, in principle, on the relative probability of infection per node. Nevertheless, the choice should be made in order to ensure that, in most simulations, the endemic status is reached (i.e. the infection does not die out). In our case, after some exploratory experiments, we selected a probability of the infection to be transmitted from one node to another (*β*) equal to 0.1, and a probability of infected nodes to reverse their condition back to susceptibility (*λ*) equal to 0.8.

Each SIS model started from a random small infection focus of 61 neighboring nodes (i.e. 0.1% of all nodes in the network). The model was then allowed to run until the infection became endemic or, in a few cases, died out. We then recorded the probability, *ρ*, of each node to be infected at a given moment in the simulation^17^. The simulations were run on a slightly modified version of the network where we ensured connectivity. This step was important to avoid biases in the computation of the relative importance of nodes as spreaders. In fact, in a disconnected network, the probability of a node to be infected at a given time is necessarily equal to 0 in all clusters different from the one where the epidemic started. In turn, the probability of a cluster to include the focus is proportional to its size. Therefore, if the network is disconnected, in most simulations all nodes in clusters other than the largest connected one would not be reached by the infection, which would prevent a fair evaluation of their importance as potential spreaders.

To make the network connected we looked for the most parsimonious solution, i.e. that minimizing both the number of edges to be added to the original network, and the total distance covered by those edges. For this, we (i) identified all the links connecting the pair of closest nodes between any two clusters. In doing this, we took into account also all isolated nodes that were not included in the 1 km network. Then we (ii) sequentially tested removing those links one by one, in decreasing order starting from those connecting the farthest orchards to those connecting the closest ones. At each attempt, we accepted the removal only if it did not disconnect the network. This procedure led to the identification of a relatively small list of candidate edges to be added to the 1 km network in order to make it connected. Starting from this set of candidates, we (iii) used a genetic algorithm to either reduce its length, or the corresponding total distance in the geographical space. The algorithm tried iteratively to replace candidate edges with some random edges from those identified in (i) or to remove edges, retaining those mutations not breaking connectivity and leading to a combination of edges corresponding to a shorter overall distance in the geographic space. We let the algorithm run until 100,000 consecutive mutation attempts did not show improvements. The resulting connected network contains 316 additional edges (for a total of 870,097) as well as 149 additional (previously isolated) nodes, for a total of 61,035. The average distance between the two nodes selected by the algorithm to connect different clusters was 1443 ± 521 m (minimum 1001 m; maximum = 4674 m) (Fig. S6).

One may argue that the disconnection of a cluster from the largest connected component implies immunity for that cluster, and that it is therefore correct to consider all nodes in the cluster as very poor spreaders. Nevertheless, we decided to connect the network before running the SIS simulations for various reasons. First of all, the aim of our analysis was to provide a general evaluation of spreading importance for all nodes. Second, the criterion we used to connect the isolated clusters to the LCC, i.e. the addition of a minimum number of edges, still ensures a strong separation between the original different clusters, i.e. the probability of the infection to remain within a certain cluster will depend substantially on the cluster’s structure. Third, even if our approach is by no means intended to be a realistic spread simulation (as explained above, SIS were used because they provide a functional estimate of *ρ*), it is certainly unrealistic to assume that clusters of orchards separated from the LCC or from other clusters by distances moderately above our arbitrary threshold of 1 km are completely ‘immune’ to the infection. In this context the addition of edges between the closest points of separated clusters represents in a very conservative way the possibility of sporadic ‘jumps’ of the pathogen.

We ran all 1,000 SIS for 100,000 steps. Given the number of infected nodes at each *i*-th simulation step (*Ni*), we identified the time needed for the infection to reach endemic status (i.e. a stable condition where the number of nodes passing from *S* to *I* equals that of nodes passing from *I* to *S*) as the *i*-th step satisfying the condition $m\le {n}_{j}\le M {\rm{\forall }}j\in [i,i+1000]$, with *m* and *M* being, respectively the minimum and maximum *N*_*k*_, with $k\in [i+1001,i+2000]$. Such condition was reached, on average, after 2,102 ± 1,308 (s.d.) steps.

In 6 cases the infection died out before the end of the simulation. In all other cases (i.e. 99.4%), the infection became endemic, with an average number of nodes infected in the last 1,000 steps of each simulation equal to 19,916 (s.d. 7712), permitting us to compute *ρ*_*i*_ for each node in the network as the frequency of steps (between 10,000 and 100,000) in which the *i*-th node was in the *I* status. The *ρ*_*i*_ values of each node were then averaged between the 994 simulations where the endemic status was reached, and rescaled between 0 and 1 in order to quantify the relative importance of orchards as potential epidemic spreaders.

As we have explained, the choice of the infection and recovery probability, in principle, should not affect the results of simulations. However, we tested this empirically by replicating all the experiments using two alternative scenarios. In one of them we simulated much weaker epidemics by setting *β* = 0.05 and *λ* = 1, while in the other one we simulated much stronger epidemics by setting *β* = 0.2 and *λ* = 0.5. The individual node *ρ*_*i*_ values were very consistent between the three scenarios, with Spearman’s rank correlation coefficients of pairwise comparisons larger than 0.8 in all cases (and *p* always <2.2e-16). Thus, we reported the results only for the intermediate scenarios with *β* = 0.1 and *λ* = 0.8.

### Why the scenario depicted by networks is conservative

We have treated the network of olive orchards as a map of the potential pathways of spread for *Xylella fastidiosa*. The basic assumption behind this network approach is that the pathogen can spread from a field to another one only within a given distance threshold μ (1 km in our case). This constrains the paths a pathogen can follow to the existing links in the network. From an epidemiological perspective, the distance between two orchards *A* and *B* measured in terms of shortest path length, i.e. the minimum number of edges leading from orchard *A* to orchard *B*, may reflect the speed of pathogen spread better than the actual linear geographical distance between *A* and *B* does^24^. In fact we may imagine both a situation where the shortest network path between *A* and *B* consists of a few intermediate nodes distributed along the ideal shortest line connecting *A* and *B* in the geographical space, and a situation where the shortest path in the network involves many nodes describing a much more curvy trajectory. Clearly, the former situation will lead to a much faster spread than the latter.

In the olive orchard network, the average minimum path distance between any two nodes is relatively large (60.5 ± 37.4 S.D., computed on a random sample of 10^4^ pairs of nodes belonging to the same component; Fig. S7), indicating that, in absence of ‘jumps’ between nodes farther away than the threshold distance, the speed of the spread could not be extremely fast.

First reports of the olive quick decline syndrome, which has been associated with *X. fastidiosa* infection, came from the region near Gallipoli between 2008 and 2010, as Martelli *et al*.^3^ suggested in 2016. Starting from the first orchard (Donato Boscia, personal communication) where the infection was detected (40.0177° N, 18.0561° E)^29^, reaching the westernmost infections of *X. fastidiosa* (40.5030° N, 17.6137° E; reported on 30/03/2015)^21^ requires a minimum of 34 steps when moving from one orchard to another through the 1 km network. Assuming *X. fastidiosa* started spreading in 2010, this equates to 6.8 steps/year.

Moving through the network from the first known infected orchard to the northernmost orchard reported in the high resolution map (41.9448° N, 16.0438° E) requires a minimum of 296 steps. The empirical data suggests that it would take *X. fastidiosa* 296/6.8 = 43.5 years to cover this distance (the same computation gives 62.8 years for the network with μ = 500 m). To replicate this rate of spread through our network, requires that the latency lapse between the time of infection of a plant and the transmission to other plants is unrealistically short, or the probability of infection is much higher than implied in our model (Supplementary Fig. S8). For example, if the incubation time were only 10 days, the daily probability of the pathogen passing from an infected field to a neighboring one would have to exceed 0.45 over the 5-month period that the vector *Philaenus spumarius* is active^10^. Moreover, the estimate of 43.5 years creates an upper boundary for the number of incubation days (at around 20), since probability of infection cannot exceed 1. For the network with μ = 500 m, the minimum transmission probability increases to 0.59 when incubation time is 10 days, and the upper boundary for the incubation days is set to 17 days.

We computed the average number of steps *X. fastidiosa* is moving per year by assuming that the probability of infection will not increase with time. This, however, could be unrealistic, due to the fact that the probability of an orchard to be infected will increase as the pathogen spreads within or between orchards. If the speed of *X. fastidiosa* spread increases with time, our prediction of the time needed by the pathogen to spread from the first known infected orchard to the northernmost one would be less than 43.5 years. In turn, this would make the dotted line in Supplementary Fig. S8 steeper, setting an even smaller, less realistic^30^ upper boundary for the incubation time.

For the network to allow for the simulations of the observed rate of spread under reasonable estimates of incubation time and infection probability, the threshold distance for the dispersion of *X. fastidiosa* (μ), needs to be increased beyond 1 km. This would lead to a more densely connected, and resilient, network that would require even greater effort to break down. Our results based on the 1 km network therefore represent a best-case scenario.

### Software used

We performed all the analyses using self-coded Python^31^ scripts and drawing from Python packages. For network analysis we relied on the package *Igraph* (http://igraph.org/python), while all the maps were realized using *Basemap* (http://matplotlib.org/basemap). The network plot (Fig. 1) was realized using the free software Gephi (https://gephi.org/). The shapefiles of olive orchards distribution and of management zones were processed using the packages *Fiona* (https://pypi.python.org/pypi/Fiona) and *Shapely* (https://pypi.python.org/pypi/Shapely). Graphs were realized using *R* ^32^ and the *Python* package *Matplotlib* (http://matplotlib.org).

## Electronic supplementary material

Supplementary Information
