# Supplementary material for: Network analysis reveals why Xylella fastidiosa will persist in Europe
Source: Sci Rep. 2017 Mar 6;7:71. doi: 10.1038/s41598-017-00077-z (PMC5428346; doi:10.1038/s41598-017-00077-z)
Supplement: Supplementary file 1 — Supplementary Information [file 41598_2017_77_MOESM1_ESM.pdf]

Supplementary Information

Network analysis reveals why *Xylella fastidiosa* will persist in Europe

Giovanni Strona[1*], Corrie Jacobien Carstens[2] & Pieter S.A. Beck[1]

1 European Commission, Joint Research Center, Directorate D - Sustainable Resources, Bio-Economy Unit, Via Enrico Fermi 2749, 21027 Ispra (VA), Italy

2 School of Mathematical and Geospatial Sciences, RMIT University, Melbourne, Victoria 3000, Australia

*Correspondence to: goblinshrimp@gmail.com

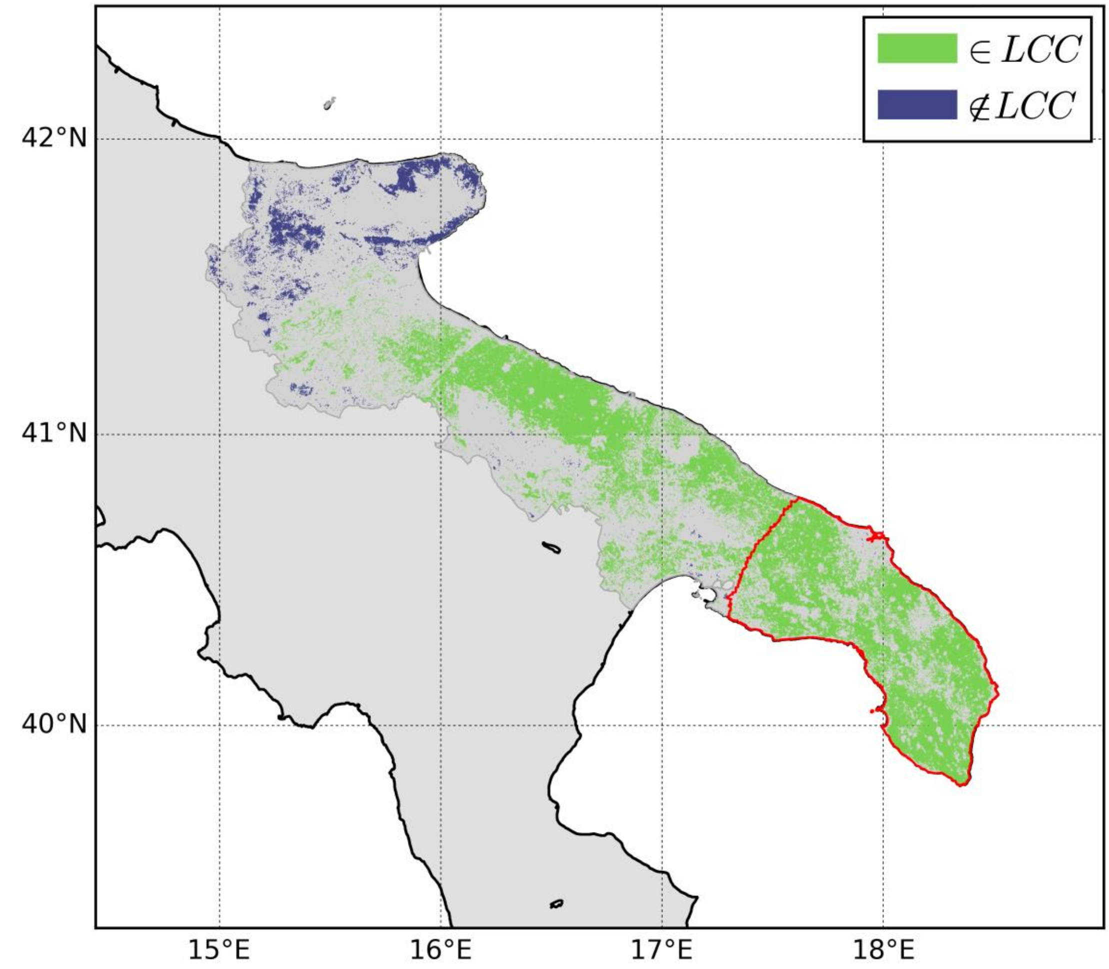

Supplementary Figure S1 | The largest connected component (LCC) in the olive orchard network (μ = 1 km) in Italy's Puglia region. Orchards in green belong to the LCC, while those in blue do not. The red line indicates the perimeter of the infected area in the current *Xylella fastidiosa* outbreak[11]. The map was generated using the Python Basemap Matplotlib Toolkit (http://matplotlib.org/basemap/).

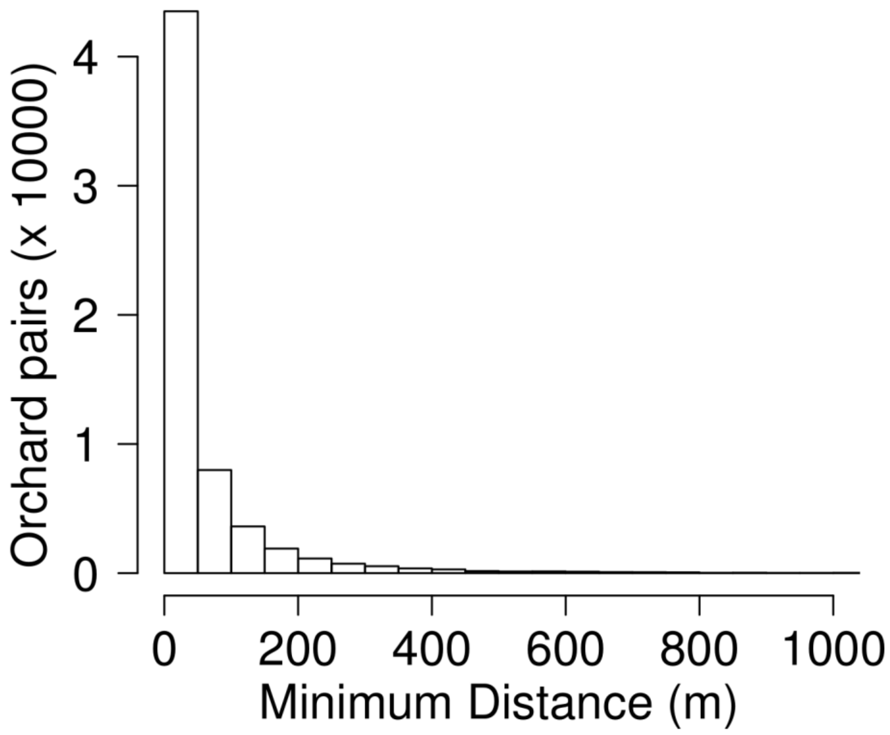

Supplementary Figure S2 | Frequencies of the spatial distances between any node in the network and its nearest neighbor. Distance between two polygons (i.e. orchards) was measured as the shortest straight line that can connect the perimeters of the two polygons.

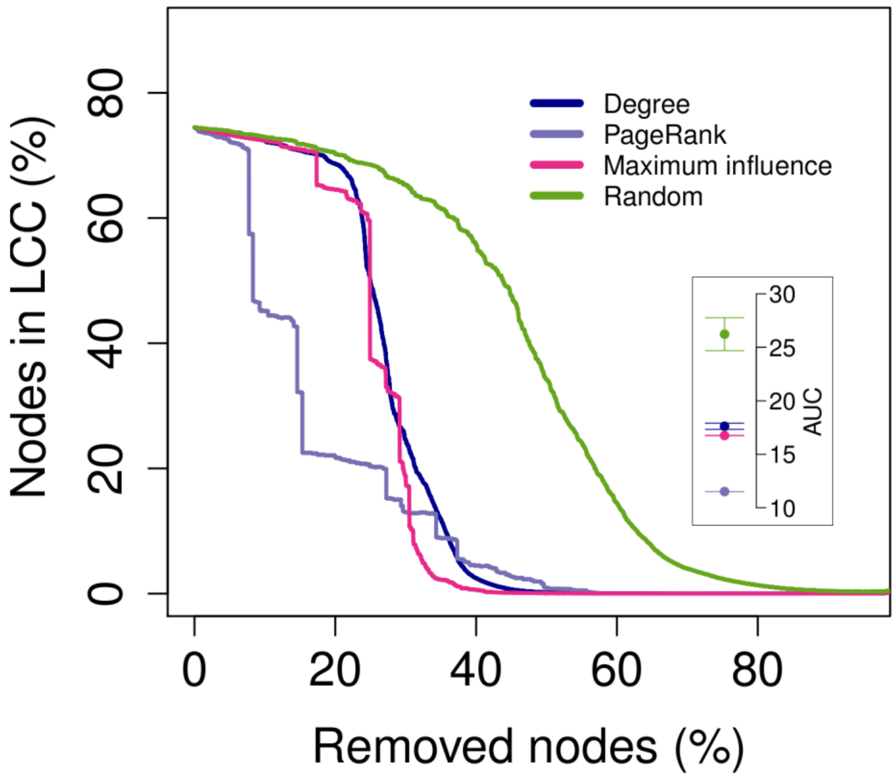

Supplementary Figure S3 | Effort needed to immunize the olive orchard network obtained using a threshold μ = 500 m. The graph shows the fraction of nodes remaining in the largest connected component (LCC) of the olive orchard network after progressive node removal according to different criteria (see Material and Methods). The solid lines interpolate the average values of 100 replicates. The inner box reports the average areas under the curve (over 100 replicates) for each removal criterion, rescaled between 0 and 100, with error bars indicating standard deviations.

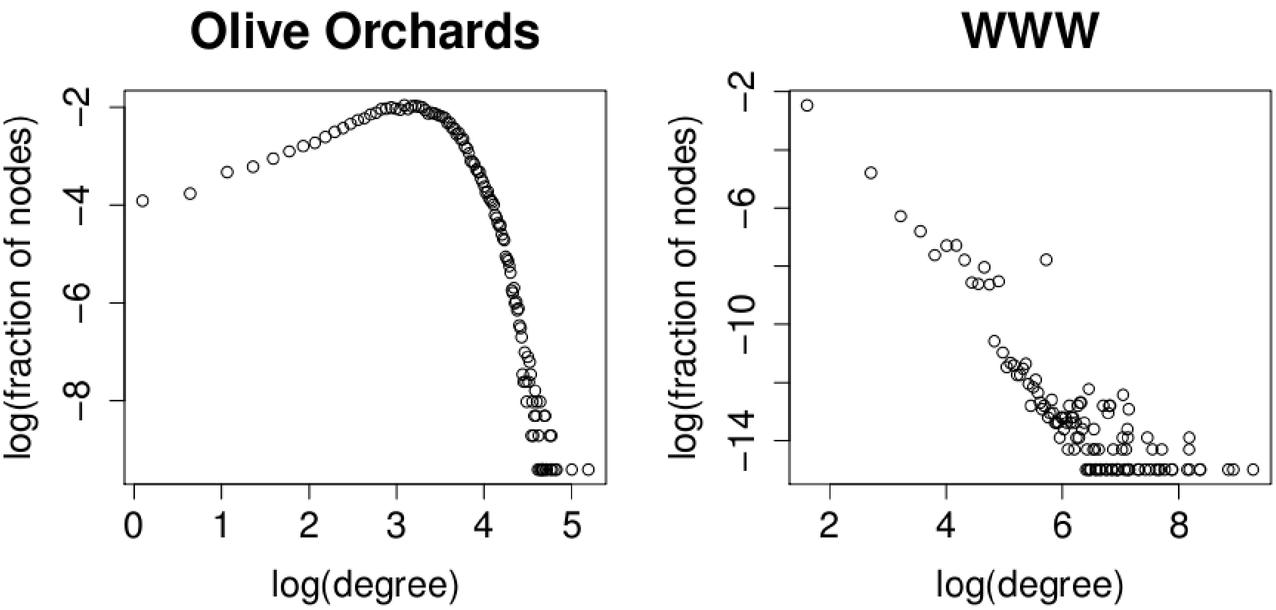

Supplementary Figure S4 | Degree distribution of the network of olive orchards compared to that of a scale free network. The distribution of node degree (i.e. the number of connections per node) in the network of olive orchards (left panel) is substantially different from the power law distribution observed in many real-world network, such as the WWW[21] (right panel). In particular, it has a high fraction of nodes with a moderately high degree, which complicates both immunization and prioritization.

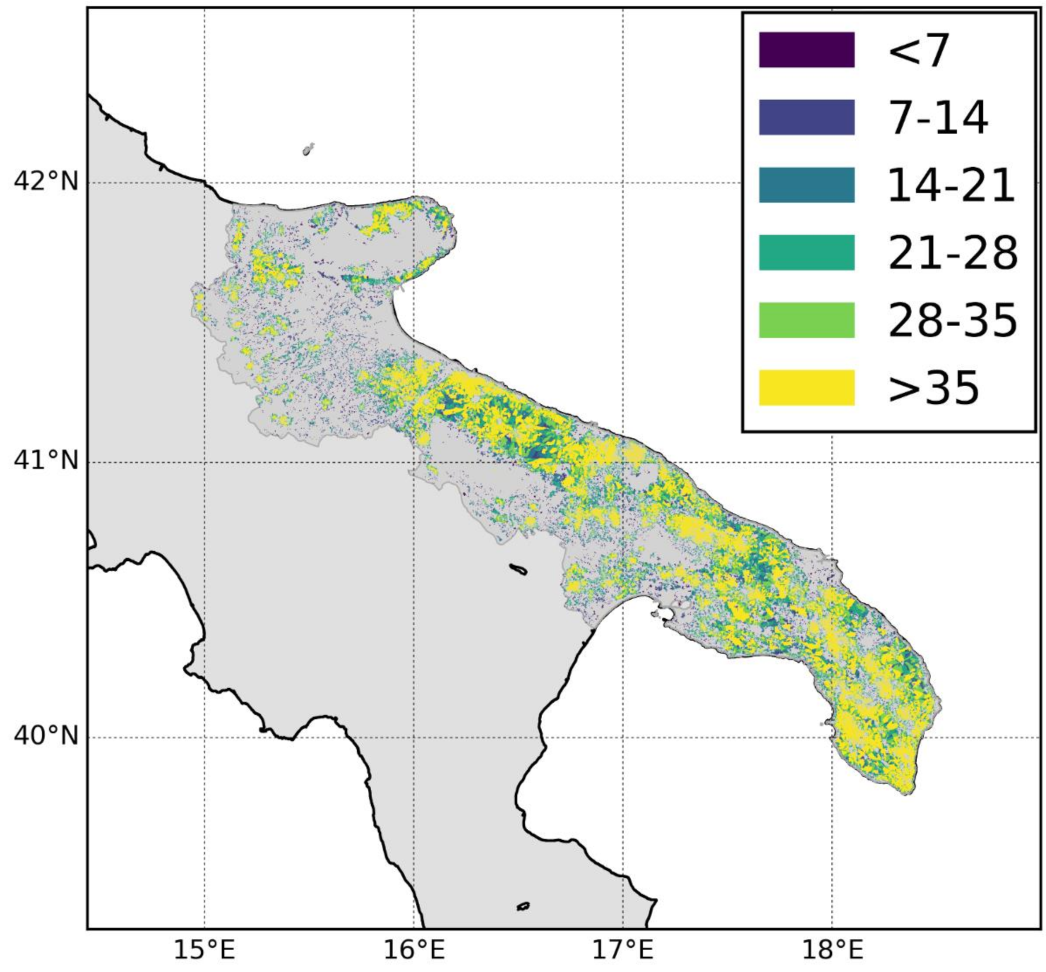

Supplementary Figure S5 | Geographical distribution of node degree, i.e. number of connections per orchard. The degree distribution is uniform across the region, with most orchards having more than 35 connections. The map was generated using the Python Basemap Matplotlib Toolkit (http://matplotlib.org/basemap/).

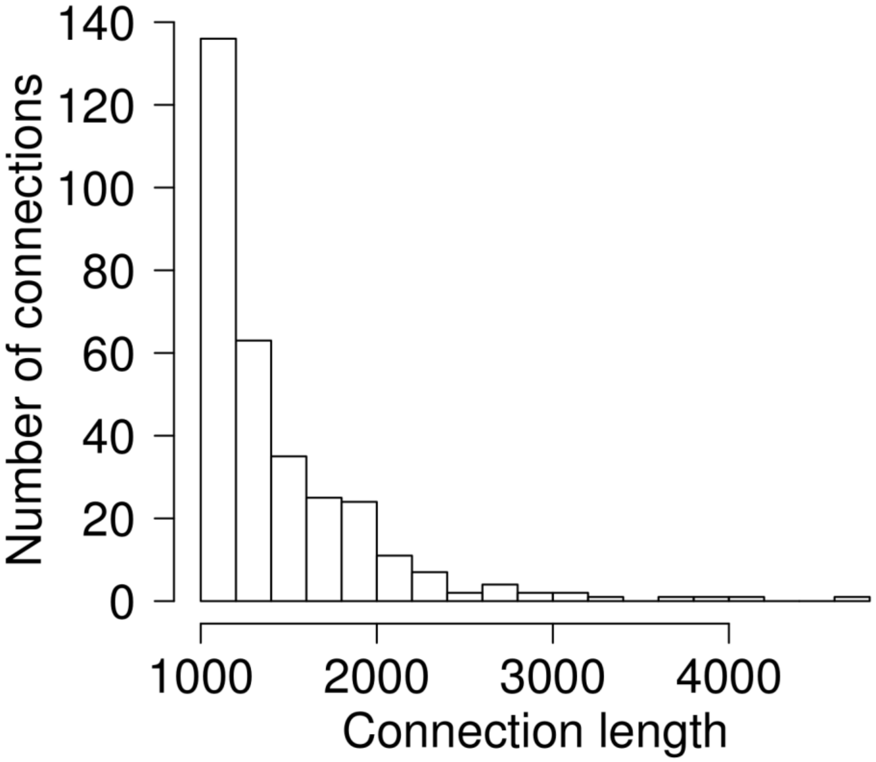

Supplementary Figure S6 | Frequencies of the length of connections used to make the olive orchard network fully connected. The connections needed to join separated components of the network into a single connected component were identified using a genetic algorithm aimed at minimizing the cumulative length of the additional connections (see **Methods**). Distance between two polygons (i.e. orchards) was measured as the shortest straight line connecting the polygons' closest sides.

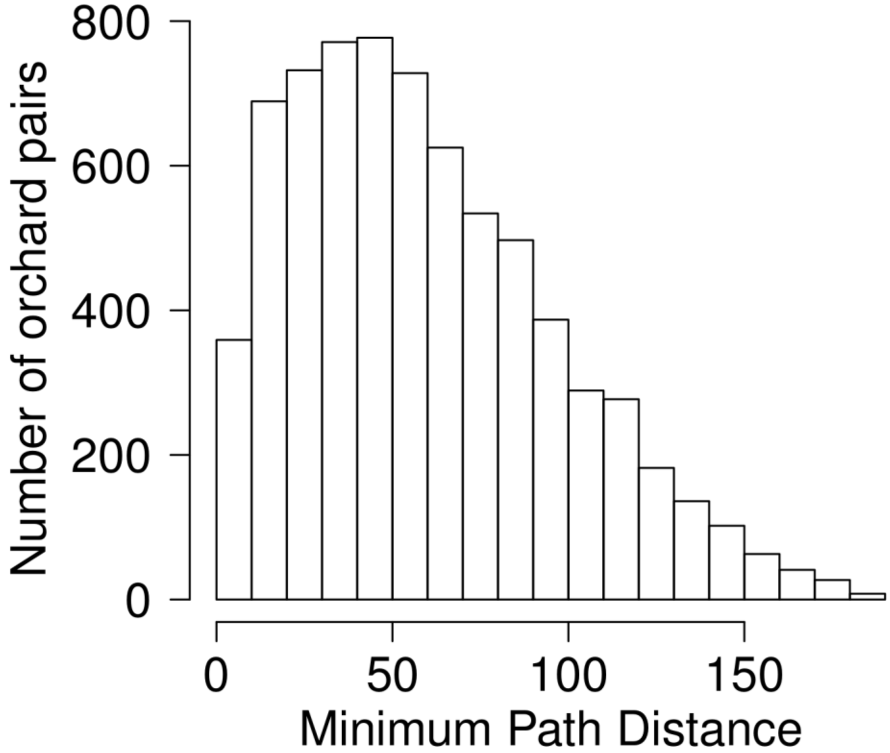

Supplementary Figure S7 | Frequencies of the minimum path lengths between any two nodes in the network. Frequencies were estimated from a random sample of 10000 node pairs.

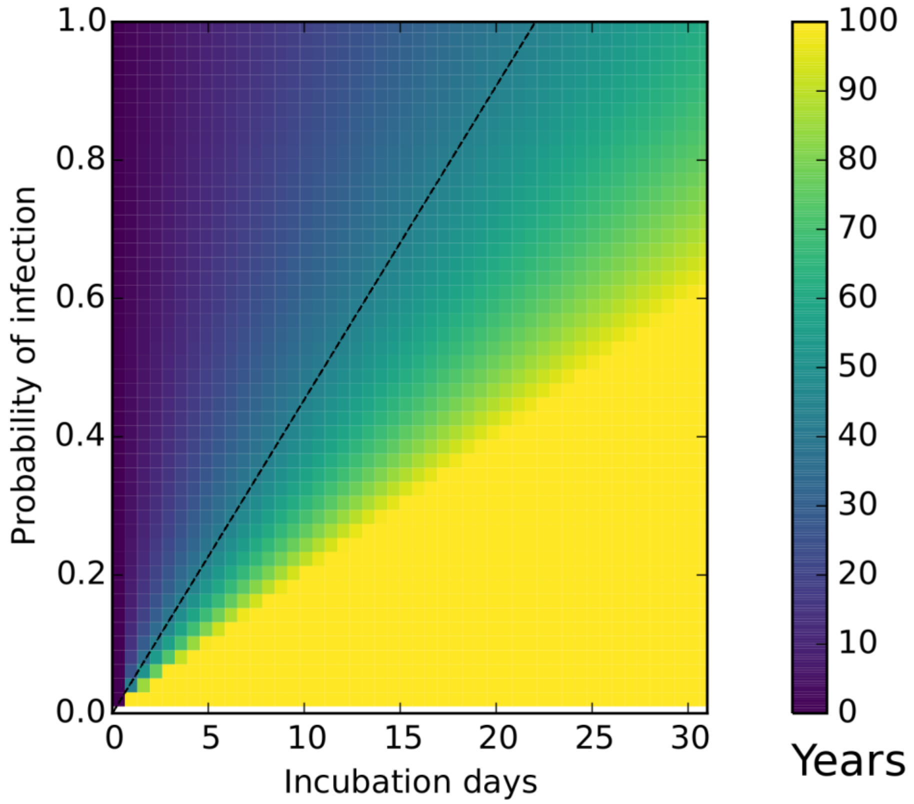

Supplementary Figure S8 | Hypothesized time required for *Xylella fastidiosa* to spread across Puglia as a function of incubation time and probability of infection. Colours indicate the time (t) required for *X. fastidiosa* to move from the origin of the infection, around Gallipoli, to the northernmost orchard in Puglia, through the network (μ = 1 km). It was calculated as t = (D × incubation days) / (probability of infection × 150), with D = 296, representing the distance (in terms of steps in the 1 km network) from the origin of the infection to the northernmost orchard in the region. The value 150 indicates, in days, the annual activity period of the vector *Phylaenus spumarius*. Bright yellow areas represent values longer than 100 years. The dotted line indicates the combination of incubation days and probability of infection values leading to a t = 43.5.
